# Medical Items

**Published:** 1917-02

**Authors:** 


### MEDICAL ITEMS

### PROMOTING TISSUE RESISTANCE IN CHRONIC BRONCHITIS.

A valuable therapeutic point in the treatment of chronic bronchitis, and particularly the bronchitis of the aged, is the aid given by tissue reconstructives. Thus, Cord· Ext. Ol. Morrhuae Comp. (Hagee) administered systematically to this class of patients exerts a marked effect on the bronchial condition, reducing the secretion and irritation, largely through its general reconstructive force. The bronchial and pulmonary tissues' resisting power—that is the power to throw off infection—is augmented, an effect shown by improvement in the cough, irritation and amount and character of the secretion·

---

### PASADYNE IN HYPERCEREBRATION.

For the purpose of reducing irritability of the higher centres, as manifested by restlessness and overstimulation of the mental processes, PASADYNE (Daniel) possesses exceptional value. Merely a ditinctive concentrated tincture of passi__ora incarnata (a time tried cerebral sedative) PASADYNE (Daniel) is safe and dependable and has the great adwithout evil after-effects. Try it as a sleep producing agent. vantage that it may be given freely to women and children A sample bottle may be had by addressing the laboratory of John B. Daniel, Inc., Atlanta, Georgia.

---

IN PREGNANCY where elimination is deficient, as indicated by headache, slight disturbance of the digestion and diminution of solids and urea in the urine, sanmetto in connection with calomel is remarkably effective. The calomel acts upon the cells of the body, those of the liver especially, effecting proper removal of the waste and accumulated toxins. Sanmetto increases the activity of the kidneys, in this way promoting the removal of excrementitious products from the blood, and at the same time acts as a systematic tonic enabling the body to more completely dispose of its waste products through its organs of elimination, and resist the evil effects from systematic absorption of auto-toxins.

### PHYLACOGENS IN SMALL BULBS.

Formerly the six preparations constituting the Phylacogen line were supplied in 10-mil (10-Cc.) bulbs only. A considerable demand has developed for a smaller package. To meet it the manufacturers (Parke, Davis & Co.) announce the addition of a 1-mil (1-Cc.) bulb. Each bulb is enclosed in a pasteboard carton and is accompanied by a descriptive circular. These small bulbs are marketed in packages of five, which enables the druggist to supply the physician with one to five bulbs, as may be wanted. The 10-mil bulbs, in individual cartons, will be marketed as heretofore. It is confidently believed that the two packages now furnished will meet every demand of the medical profession.

---

IN INFLAMMATORY DISEASES OF THE SKIN especially where volumetric analysis shows defective urinary elimination, sanmetto will be found a useful remedy, owing to its direct action on the kidneys.

